# Visuomotor reaction time can predict IQ in children

**DOI:** 10.1192/j.eurpsy.2021.1301

**Published:** 2021-08-13

**Authors:** N. Kiseleva, S. Kiselev

**Affiliations:** 1 Laboratory For Brain And Neurocognitive Development, Ural Federal University, Ekaterinburg, Russian Federation; 2 Clinical Psychology, Ural Federal University, Ekaterinburg, Russian Federation

**Keywords:** processing speed, intelligence, visuomotor reaction time

## Abstract

**Introduction:**

It is well established that reaction time and IQ test scores are correlated, although the strength of this relationship is a matter of debate (Neisser et al., 1996). It was proposed that processing speed is a component of intelligence (Deary, Penke, & Johnson, 2010; Hunt, 2011). In our previous research we have not revealed the relationship between IQ and reaction time in children (Kiselev et al., 2000). However, it is possible that reaction time can predict intelligence test scores in the developmental perspective.

**Objectives:**

This study investigated whether visuomotor reaction time in 5 year-old children predicts intelligence test scores in 8 year-old children using the longitudinal approach.

**Methods:**

The participants were 35 children (17 males and 18 females) at the age of 5 years (5,34±0,45). We used computerized sensorimotor technique (Kiselev et al., 2009) to investigate visuomotor reaction time in children. Children completed simple, discrimination and choice reaction time tasks. The IQ of 8-year children was assessed by the WISC.

**Results:**

The regression analysis has revealed the significant (p≤0,05) relationships between discrimination and choice reaction time tasks in 5 years-old children and non-verbal IQ performance in these children at 8 years of age. However, we did not find this relationship for simple reaction time task.

**Conclusions:**

In view of obtained results it can be assumed that visuomotor reaction time in preschool children can predict non-verbal intelligence test scores in the developmental perspective. The received data can give new perspective in the understanding the interrelation between reaction time and IQ in children.

